# Why Early Cancer Is Curable

**Published:** 1917-05

**Authors:** 


### Why Early Cancer is Curable.

There is still a widespread misapprehension that cancer is a constitutional disease caused by some substance or poison in the blood. Those who hold this mistaken opinion commonly believe that the disease is hereditary, and in a vague way they think there must be some taint handed down from one generation to another which causes cancer to flourish in certain families. In the minds of people not well informed on the subject this belief may well cause a feeling that it is somehow shameful to have the disease. Such misapprehension, combined with the notion which has long prevailed that cancer is a hopeless, incurable affliction, and that it is of no use to try to have anything done for it, may well account for the extraordinary delay of many sufferers in seeking treatment. A further cause is the fact that cancer, in the early stages, often causes little or no pain. Many a surgeon has wished that cancer, in its early manifestations, might cause the sufferer half as much trouble as a toothache, for then the patient would surely be driven to seek relief so quickly that he or she would be easily cured.

That cancer is at first a local growth and not a general disease of the system is now clearly established. This fact is of the utmost importance, since it holds out a high hope of cure if the malignant growth is removed before it has time to spread to other parts of the body. Cancer beginning in one spot later appears elsewhere, because small particles or cells are carried away from the first site and start other growths, not because there exists previously some poison in the blood which causes the disease to break out in different parts of the body. The great hope of cure, therefore, lies in removing cancer entirely from the system before it has a chance to spread from its first foothold.

The reason why so many people came to believe that cancer was a blood disease is doubtless because it was observed to come again in the same or other parts of the body after having been apparently cut out. It was natural to assume that when the disease kept coming back in this manner there must be some cause or taint in the blood which led to its breaking out in different places much like certain skin diseases. The trouble which started this fallacious reasoning was that in those earlier days cancer was not so well understood as it now is. Surgeons then did the best they knew how, but without the advantages of modern methods they were unable successfully to exterminate the disease. The microscope has now shown us the paths by which cancer cells start their invasion of the body if the first and local appearance is neglected. Modern surgeons are, therefore, repeatedly successful in removing the disease once for all. As an eminent American doctor has well said, "It is not surgery, but *delayed* surgery that fails to cure."—*Clipped.*

